# Engineering Surface and Optical Properties of TiO_2_-Coated Electrospun PVDF Nanofibers Via Controllable Self-Assembly

**DOI:** 10.3390/nano8090741

**Published:** 2018-09-19

**Authors:** Jianming Yang, Fuan He, Huijun Wu, Yuying Liang, Yuxuan Wang, Zhi Sun

**Affiliations:** 1 College of Civil Engineering, Guangzhou University, Guangzhou 510006, China; y1224588051@163.com (J.Y.); liangyuying.y@foxmail.com (Y.L.); wangyuxuan_berg@163.com (Y.W.); sunzhiwait13@163.com (Z.S.); 2 College of Chemical Engineering, Guangdong University of Petrochemical Technology, Maoming 525000, China; he-fu-an@163.com

**Keywords:** TiO_2_, PVDF, electrospinning, self-assembly, extinction, refractive index

## Abstract

Understanding the effect of a porous TiO_2_ nanolayer on the optical scattering and absorption through electrospun fibers is of great importance for the design and development of advanced optical extinction materials. Based on electrospinning and controllable self-assembly techniques, pure electrospun poly(vinylidene fluoride) (PVDF) fibers and TiO_2_-coated ones with different self-assembly cycles were prepared. The effect of TiO_2_ self-assembly cycles on surface parameters, e.g., thickness, assembled content, and porosity of the TiO_2_ nanolayer were determined by scanning electron microscopy, thermogravimetric analysis, and Fourier transform infrared spectroscopy. With an increase in the self-assembly cycles, the TiO_2_-coated electrospun PVDF fibers presented rougher surfaces and greater average diameters. According to the characterized surface parameters, the effects of the controllable self-assembly on the optical refractive index, absorption index, and infrared extinction were investigated to increase the optical properties of electrospun PVDF fibers. The results indicated that an increase of almost 120–130 cm^−1^ in infrared extinction could be achieved through the controllable self-assembly with only 5.7 wt. % assembled TiO_2_ content. This is highly efficient when compared with other coating modes. We believe that this study could give some positive guidance in the design of TiO_2_-coated electrospun fibers for improving their surface and optical properties.

## 1. Introduction

Fibrous materials, given their excellent optical extinction capacity, have drawn growing attention in both academic [1,2] and industrial areas [3], and mainly include commonly used fibers and electrospun fibers [4]. Electrospun fibers have remarkable characteristics such as a fairly large surface-to-volume ratio, high porosity, low density, and excellent flexibility [5,6]. In general, electrospun fibers can be cataloged into pure organic fibers, pure inorganic fibers, and organic/inorganic hybrids [7] such as poly(vinyl alcohol) (PVA) [8] for organic fibers, carbons [9] for inorganic fibers, and polyacrylonitrile (PAN)/AgCl composite fibers for hybrids [10].

However, electrospun fibers generally exhibit a lower optical extinction capacity than the commonly used fibers because of their much smaller diameters [11]. For instance, Caps et al. [12] studied the influence of the diameter on the infrared extinction of polypropylene fibers and found that the infrared extinction decreased from ~50 to 10 m^2^·kg^−1^ as the diameter of polypropylene fibers decreased from 6 to 1 μm. Nanoparticles with excellent capabilities of refraction and absorption (e.g., Al, SiC, TiO_2_) are generally incorporated into the polymer [13,14,15,16,17] to improve the optical extinction capacity such as electrospun PAN-Ag composites [18]. Among these nanoparticles, nano TiO_2_ presents remarkable superiorities in a large surface area, an ultrafine size, and a strong interfacial interaction [19]. For instance, the combination of TiO_2_ nanoparticles and electrospun fibers have exhibited various potential applications in protective clothing systems, photocatalysis, sensors, and electrodes [20,21].

It is obvious that the distribution of nano TiO_2_ in an electrospun fibrous membrane plays a key role in the increase of optical extinction capacity. There are five typical modes in relation to the distribution of nano TiO_2_. The first mode is a random or uniform distribution by a directly physical mixture during the preparation process [22]. Our previous study on a random distribution of TiO_2_ particles showed that the optical extinction capacity could be increased by ~40% with the addition of 10 wt. % TiO_2_ particles into the fibrous matrix [23]. The second mode is to form a smooth and continuous TiO_2_ membrane on the surface of electrospun fibers [24]. For instance, Danion et al. coated a TiO_2_ membrane on the surface of optical fibers and prepared a single-TiO_2_-coated optical fiber reactor [24]. The results indicated that as the thickness of the TiO_2_ membrane increased from 0 to ~300 nm, the extinction coefficient was increased by 3000 cm^−1^ at the axis direction. The third mode is the nanorod coating with a certain diameter and length [25]. Yang et al. prepared TiO_2_ nanorod coating layers on mullite fibers for infrared opacifier application [26]. They found that the optical properties of mullite fibers could be improved up to 2–4 folds after the coating of TiO_2_ nanorod layers. The fourth mode is the rice-like geometry of the TiO_2_ nanoparticles coated on the surface of electrospun fibers. For example, Zhao et al. [27] prepared nylon 6 fibers with a TiO_2_ rice-like coating and investigated their extinction capacities. The results indicated a high increase of almost 200 cm^−1^ in the extinction coefficient after the TiO_2_ rice-like coatings.

Apart from those modes above-mentioned, controllable self-assembly is a particular mode that forms a porous nanolayer of TiO_2_ nanoparticles coated on the surface of electrospun fibers [28]. Layer-by-layer (LBL) self-assembly has been widely considered as one of the most popular coating techniques that involves the sequential adsorption of oppositely charged materials to construct ultrathin conformal coatings [29]. This coating mode enlarges the scattering and absorptive influences given its porous nanostructure, and thereby could be an efficient strategy to enhance the optical extinction capacity of electrospun fibers. However, as far as we know, knowledge regarding the influence of a porous TiO_2_ nanolayer on the optical extinction capacity is extremely limited, although this influence can be significant [30].

Therefore, the aim of this paper was to experimentally investigate the engineering surface and optical properties of TiO_2_-coated electrospun fibers. By taking poly(vinylidene fluoride) (PVDF) fibers as an example of fibrous materials, TiO_2_-coated electrospun fibers were first prepared by assembling TiO_2_ on the fiber surface with the techniques of electrospinning and LBL assembling. Next, the effect of the controllable self-assembly on the optical refractive index, absorption index, and infrared extinction were investigated to optimize the optical properties of electrospun fibers. Through a comparative analysis with different coating modes and surface morphologies, the mechanism of the increase in infrared extinction of TiO_2_-coated electrospun fibers through the controllable self-assembly was revealed.

## 2. Experimental

### 2.1. Materials

PVDF particles, ethyl alcohol (EtOH, >99%), and *N*,*N*-dimenthyl-formamide (DMF, >98%) were bought from Baishi Co. Ltd., Ningbo, China. Nano TiO_2_ powder (anatase, diameter of ~7–10 nm, hydrolyzed) was bought from Jingrui New Material Co. Ltd., Xuancheng, China. Polyacrylic acid (PAA, >99%) was purchased from Damao Co. Ltd., Shanghai, China. Hydrochloric acid (HCl, ~37%) was bought from Guanghua Co. Ltd., Guangzhou, China and used by diluting with 10-fold deionized water.

### 2.2. Preparations of TiO_2_-Coated Electrospun Fibers

**Figure 1:**
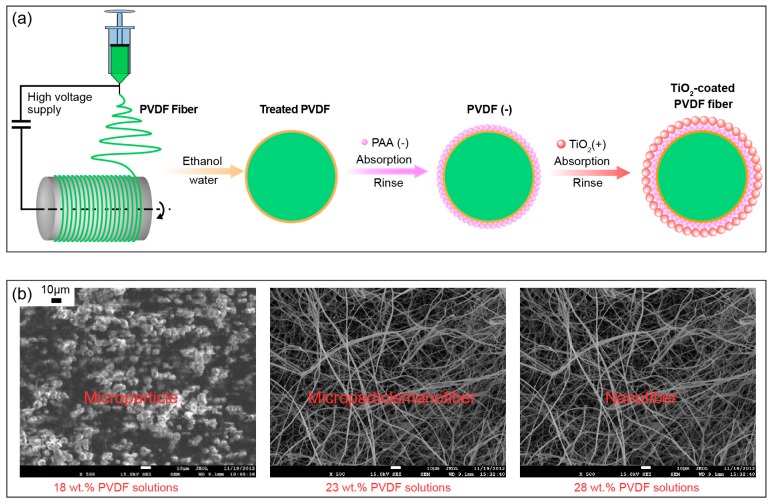
Preparations of TiO_2_-coated electrospun fibers. (**a**) Preparation process; (**b**) Influence of electrospinning parameters [34].

PVDF electrospun fibers were first produced by electrospinning [31]. Next, TiO_2_-coated electrospun fibers were prepared by using the LBL self-assembly technique [32]. Figure 1 shows the detailed preparation process with the following five steps:

(i) A PVDF solution was magnetically stirred for 0.5 h in a 60 °C water bath, and for another 0.5 h in a 25 °C water bath to cool down.

(ii) The PVDF solution was poured into a syringe in an electrospinning apparatus (Model: NEU), which was made by Kato Tech. Co. Ltd., Sangyo, Japan. The main components for electrospinning included a syringe pump, a DC power, a grounded electrode, a stainless nozzle (with a diameter of 1.0 mm), and a drum collector covered with aluminum foil to collect the electrospun fibers.

(iii) The collected electrospun fibers were dried for 24 h in an oven (model: DGX-9243B, Fuma Lab. Instrum. Co. Ltd., Shanghai, China) at 60 °C. The electrospun PVDF fibers were then treated by soaking in ethanol and replacing with water for 0.5 h to improve the wettability of water on the electrospun PVDF fibers.

(iv) The process of the coating for a porous TiO_2_ nanolayer on the electrospun PVDF membrane by one self-assembly time was as follows. An anionic PAA solution with a concentration of 10 mol. % in deionized water was prepared. An aqueous dispersion solution with a 0.5 wt. % ratio of TiO_2_ particles to deionized water was prepared with a pH of 2.5. The wetted electrospun PVDF fibers were immersed into the anionic PAA solution for 15 min and deionized water for three minutes (three times, 1 min/time) in sequence. Next, the PAA-attached electrospun PVDF fibers were soaked in the aqueous dispersion solution of TiO_2_ for 15 min and deionized water for three minutes (three times, 1 min/time) in sequence. 

(v) Step (iv) can be repeated to obtain TiO_2_-coated electrospun PVDF fibers with different thicknesses of the porous TiO_2_ nanolayer through controllable self-assembly cycles. In this paper, we prepared four samples in total, i.e., pure electrospun PVDF fibers (abbreviated as PE), TiO_2_-coated electrospun PVDF fibers with two, four, and six self-assembly cycles (abbreviated as TE#1–TE#3, correspondingly).

It should be noted that the electrospinning parameters (e.g., the solvent, solution composition, distance between needle and target, and voltage) have an influence on the morphology and fiber diameter of electrospun fibers. Compared with some other solvents such as N-methylpyrrolidone and dimethyl sulfoxide, the solvent DMF was considered to create the smallest fiber diameter and highest piezoelectric properties [33]. Furthermore, our previous studies presented the influence of solution composition (18 wt. %, 23 wt. %, and 28 wt. % solution of PVDF particles to DMF) on the morphology and fiber diameter of electrospun PVDF fibers, as shown in Figure 1b [34]. Of the three PVDF solutions, the 28 wt. % showed the optimal one, as its nanofiber was several hundred nanometers in diameter, while it showed microparticles and microparticles/nanofibers for the 18 wt. % and 23 wt. % PVDF solutions. The other optimized settings were 15 cm for the distance between the nozzle and target, and a voltage setting of 15 kV [34,35]. Therefore, the following preparations of TiO_2_-coated electrospun fibers were based on these optimal electrospinning parameters.

### 2.3. Characterizations 

A JSM-7001F field emission scanning electron microscopy (SEM) from JEOL Ltd., Tokyo, Japan was employed to observe the microstructural morphologies of the samples. All samples were dried for 12 h at 60 °C before characterization.

Nano-measurer (Version V1.2.5) software (Fudan University, Shanghai, China) for particle size distribution calculations was used to measure the diameter distributions of the samples. The average fiber diameter was fitted by the Gaussian distribution. 

A thickness gauge bought from Chenlu Co. Ltd., Ningbo, China was employed to measure the thickness of the samples. Five measurements were conducted at different positions of each sample and averaged to obtain an average thickness (*L_m_*). 

A BP211D electronic analytical balance bought from Sartorius Co., Aubagne, France was employed to measure the mass (*M*) of the samples.

A TGA400 thermal gravimetric (TG) analyzer bought from PerkinElmer Inc., Waltham, Mass., USA. was employed to measure the TiO_2_ content in the TiO_2_-coated electrospun PVDF fibers. The parameters of the TGA experiment included a heating rate of 10 °C·min^−1^, a heating temperature from 20 to 800 °C, and a nitrogen flow rate of 50 mL·min^−1^. 

A Fourier transform infrared (FTIR) spectrometer bought from Bruker Co., München, Germany was employed to measure the spectral transmittances ($\tau_{\lambda }$) under a wavenumber range of 400–4000 cm^−1^. The $\tau_{\lambda }$ is the ratio that an incident intensity passes through a sample. Two measurements were conducted at the front and reverse side of the sample and averaged to determine the $\tau_{\lambda }$.

## 3. Results and Discussion

### 3.1. Surface Properties of TiO_2_-Coated Electrospun PVDF Fibers

**Figure 2:**
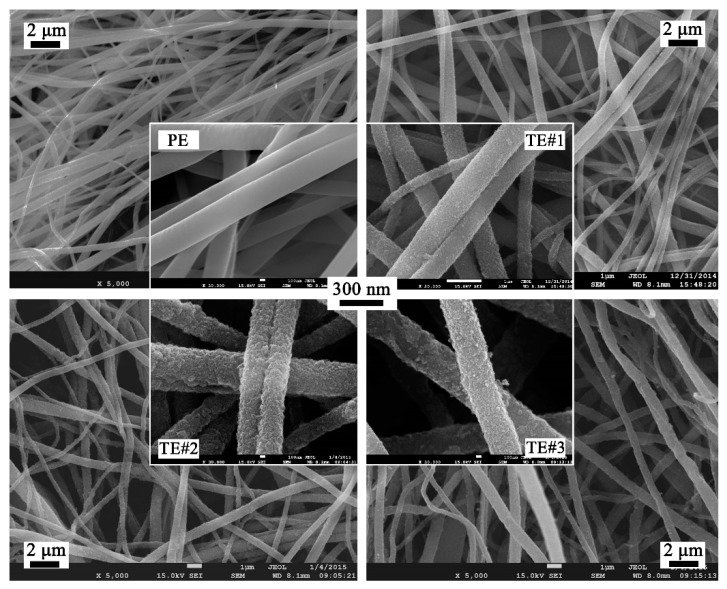
SEM images of PE and TE#1–TE#3 at magnifications of 5000 and 30,000.

Figure 2 shows the SEM images of PE and TE#1–TE#3 at magnifications of ×5000 and ×30,000. It is notable that the PE exhibited a smooth surface morphology while the surface morphologies of TE#1–TE#3 were rough. This indicates that a TiO_2_ nanolayer was successfully coated onto the surface of the electrospun PVDF fibers. According to the SEM images, an increasingly roughened surface could also be observed with an increase in the self-assembly cycles. For instance, TE#2 obtained from four self-assembly cycles showed obvious higher surface roughness than the TE#1 obtained from two self-assembly cycles. Moreover, the fibers of all samples were randomly orientated and mostly straight, and presented a long-cylinder shape with nanoscale diameters in the range of ~200–1000 nm.

Based on the SEM images, the diameter distribution could be fitted by using the Gaussian distribution as follows

(1) $$f\left(x\right)=\frac{1}{\sqrt{2\pi }\sigma_{d}}\exp\left[-\frac{{\left(x-d_{f}\right)}^{2}}{{2\sigma_{d}}^{2}}\right]$$

where f(x) represents the probability density function; x represents the normal variable; $\sigma_{d}$ represents the standard deviation of the diameter distribution from the Gaussian distribution, which indicates the uniformity of the diameter distribution of TiO_2_-coated electrospun fibers; and $d_{f}$ represents the fiber diameter.

**Figure 3:**
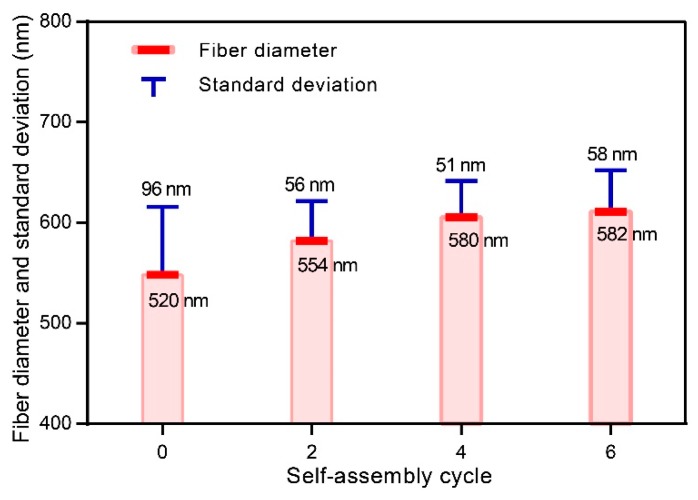
Fiber diameters and standard deviations of TiO_2_-coated electrospun fibers with different self-assembly cycles.

The average diameters of PE and TE#1–TE#3 were predicted as 520, 554, 580, and 582 nm, respectively, as shown in Figure 3. With an increase in the self-assembly cycles, the average diameter increased given that more nano TiO_2_ nanoparticles were coated onto the fiber surface while the increase rate gradually declined.

The standard deviations of the diameter distributions of PE and TE#1–TE#3 were 96, 56, 51, and 58 nm, respectively, as shown in Figure 3. It should be noted that PE had much higher standard deviations of fiber diameter than the other samples because the fiber diameter and its standard deviation are affected by the TiO_2_ assembling process. With the increase in fiber diameter, the specific surface area of the fibers decreases, which would make it difficult to deposit the nano TiO_2_ onto the fiber surface. Similarly, Kumar et al. [19] also found that the lower fiber diameter resulted in the higher surface area of the TiO_2_ nanoparticle layer. Therefore, after the TiO_2_ self-assembly process, the diameters of TiO_2_-coated electrospun fibers would get close to the average fiber diameter, which indicates a smaller standard deviation than that of the uncoated fibers (i.e., PE).

The characteristic parameters of the coated TiO_2_ nanolayer include the thickness (Th, nm), the assembly content ($L_{c}$, %), and the porosity (φ, %). Based on the fiber diameter of the uncoated fibers, the thickness of the coating layer can be determined by

(2) $$Th=\frac{d_{f}\left(n_{L}\right)-d_{f}\left(0\right)}{2}$$

where $d_{f}\left(n_{L}\right)$ represents the fiber diameter at a self-assembly cycle of $n_{L}$, then $d_{f}\left(0\right)$ indicates the fiber diameter of the uncoated electrospun fibers.

**Figure 4:**
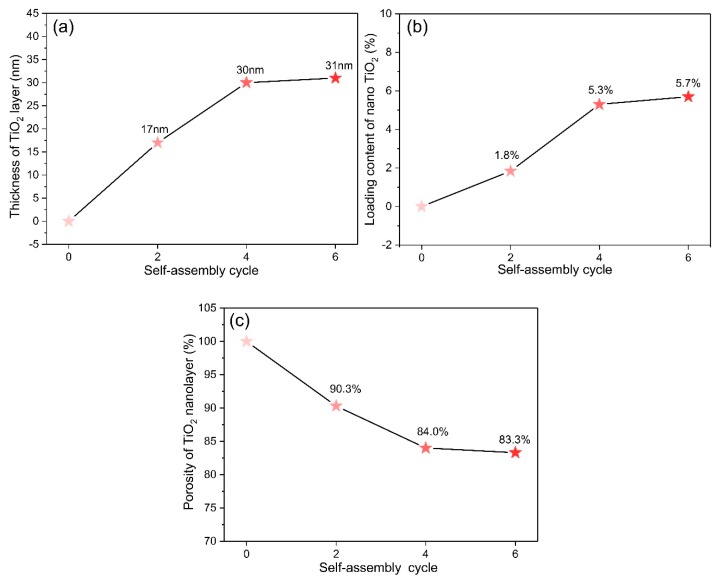
(**a**) Thickness; (**b**) assembled content, and (**c**) porosity of the TiO_2_-coated electrospun fibers with different self-assembly cycles.

The thicknesses of the TiO_2_ nanolayers of TE#1–TE#3 were 17, 30, and 31 nm, respectively, as shown in Figure 4a. It is reasonable that with an increase in the assembly cycles, the thickness of the coating layer increases, as more TiO_2_ nanoparticles can be coated on the fiber surface. Similar to the increase rate of fiber diameter, first, the thicknesses increase rapidly, then slowly with an increase in the self-assembly cycles.

**Figure 5:**
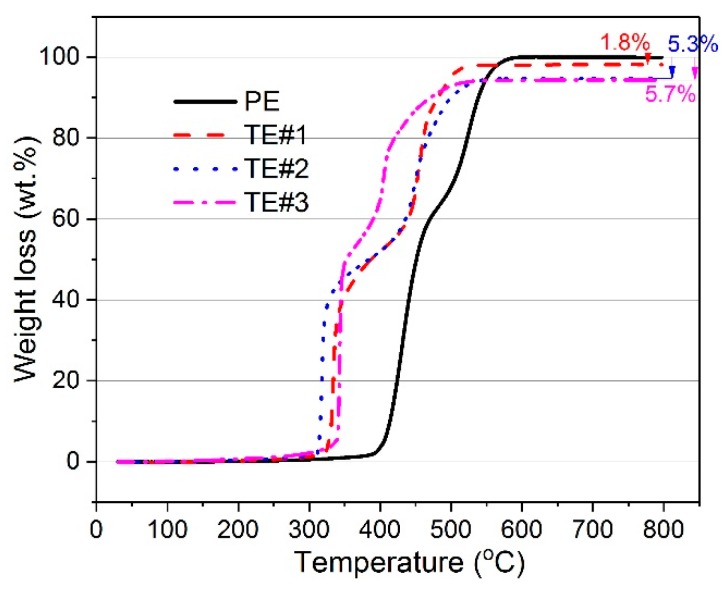
Weight loss of TiO_2_-coated electrospun fibers with different self-assembly cycles.

According to the TGA results, the weight losses of PE and TE#1–TE#3 for various temperature are shown in Figure 5. The assembled TiO_2_ content of TE#1–TE#3 were measured as 1.8, 5.3 and 5.7 wt. %, respectively, as shown in Figure 4b. It was obvious that with the increase in self-assembled layers of nano TiO_2_, the assembled content increased. However, the increase of the assembled TiO_2_ content seemed to be insignificant at only 0.4 wt. % as the self-assembly cycles increased from 4 to 6. This was much smaller than 3.5 wt. % as the self-assembly cycles increased from 2 to 4. 

The porosity of the porous TiO_2_ nanolayer can be calculated by

(3) $$\varphi =1-\frac{v_{t}}{v_{d}}$$

where $v_{t}$ is the volume ratio of TiO_2_-coated electrospun PVDF fibers to pure electrospun PVDF fiber and $v_{d}$ is the volume ratio of the TiO_2_ nanolayer to the TiO_2_-coated electrospun fibers. $v_{d}$ and $v_{t}$ can be calculated, respectively, as follows

(4) $$v_{d}=\frac{d_{x}^{2}-d_{i}^{2}}{d_{x}^{2}}$$

(5) $$\nu_{t}=\frac{\left(m_{T}/m_{P})\cdot (\rho_{P}/\rho_{T}\right)}{1/\left(1-\nu_{d}\right)}$$

where $d_{x}$ (x = 2, 4 and 6) is the diameter of the TiO_2_-coated electrospun PVDF fibers; $d_{i}$ is the initial diameter of the pure electrospun PVDF fibers; $m_{T}/m_{P}$ is the ratio of TiO_2_ mass to pure fiber mass; and $\rho_{P}/\rho_{T}$ is the ratio of TiO_2_ density to pure fiber density. The density of the TiO_2_ and PVDF fibers were 2.5 and 1.78 g·cm^−3^, respectively. 

**Figure 6:**
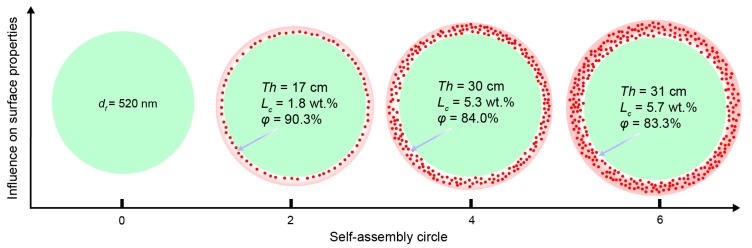
Assembly process with an increase in self-assembly cycles.

The porosity of the TiO_2_ nanolayer for TE#1–TE#3 was predicted as 90.3%, 84.0%, and 83.3%, respectively, as shown in Figure 4c. It indicates that the TiO_2_ nanolayer coated on the fiber surface had a porous nanostructure. The porosity decreased with an increase in the self-assembly cycles as further assembly on the coated fibers exerts an influence on its previous coating, as shown in Figure 6. This indicates not only an increase in the thickness perpendicular to the fiber axes, but also an increase in the space density and thereby a decrease in the porosity. This can also be seen from Equations (3)–(5) where $v_{d}$ decreases with an increase in $d_{x}$ while $\nu_{t}$ increases with an increase in $m_{T}$. Apparently, the overall φ decreases with the increase of controllable self-assembly cycles.

### 3.2. Optical Scattering and Absorption Property of TiO_2_-Coated Electrospun Fibers

**Figure 7:**
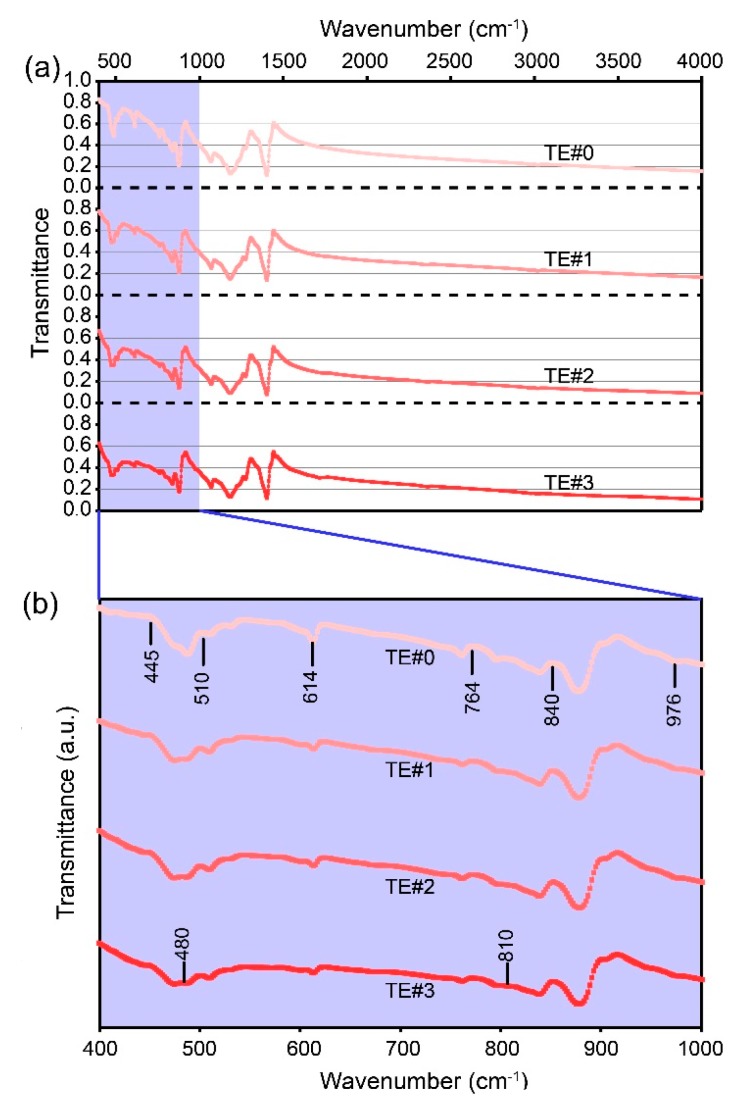
Transmittances for different self-assembly cycles. (**a**) At wavenumbers 400–4000 cm^−1^; (**b**) 400–1000 cm^−1^.

Figure 7a shows the spectral transmittances of PE and TE#1–TE#3 for various wavenumbers ranging from 400 to 4000 cm^−1^. It can be seen that the transmittances decreased with an increase in the self-assembly cycles because nano TiO_2_ has strong absorption and scattering capacities towards infrared light [19]. According to optical theories, the sum of the transmittance, absorption, and scattering ratio is 1. This indicates that the electrospun PVDF fibers possessed a higher absorption ratio and/or refractive ratio after the TiO_2_ self-assembly process since they presented lower transmittances.

The transmittances of PE and TE#1–TE#3 presented significant fluctuation in the wavenumber range of 400–1000 cm^−1^, as shown in Figure 7b. Compared with the uncoated electrospun fibers, the TiO_2_-coated ones presented a slight wave crest. The occurrence of bands at 445, 510, 614, 764, 840, and 976 cm^−1^ indicated the presence of a mixed *α* and *β* PVDF crystalline phase in the electrospun fibers. The occurrence of bands at 480 and 810 cm^−1^ can be attributed to the nano TiO_2_.

Based on the measured transmittances ($\tau_{\lambda }$), two equations for predicting the spectral absorption index ($\kappa_{\lambda }$) and spectral refractive index ($n_{\lambda }$) can be expressed by [36,37]

(6) $$\frac{\lambda }{\pi d_{f}}Re\left[\left(a_{0}+b_{0})+2\overset{\infty }{\underset{n=1}{\sum }}(a_{n}+b_{n}\right)\right]=-\frac{\pi d_{f}In\left(\tau_{\lambda }\right)}{4L_{m}\upsilon_{f}}$$

(7) $$n\left(\lambda \right)=n_{i}\left(\lambda_{i}\right)+\frac{2\left(\lambda_{i}^{2}-\lambda^{2}\right)}{\pi }P\int_{0}^{\infty }\frac{\lambda_{0}\kappa \left(\lambda_{0}\right)}{\left(\lambda^{2}-\lambda_{0}^{2}\right)\left(\lambda_{i}^{2}-\lambda_{0}^{2}\right)}d\lambda_{0}$$

where Re represents the real part of a plural; λ represents the wavelength and equals the reciprocal of wavenumber ($\sigma_{w}$, cm^−1^); $a_{n}$ and $b_{n}$ represent the Lorenz-Mie coefficients [38] that are determined by $\kappa_{\lambda }$ and $n_{\lambda }$; $L_{m}$ represents the thickness of the fibrous membrane; $\lambda_{i}$ represents the reference wavelength (e.g., 0.5 μm). Based on our previous investigation on $\kappa_{\lambda }$ and $n_{\lambda }$ of ultrafine fibers [35], the solution can be ensured to be unique as TiO_2_-coated electrospun fibers have average diameters of several hundred nanometers.

Table 1 gives the $\kappa_{\lambda }$ and $n_{\lambda }$ of PE and TE#1–TE#3 for wavenumbers ranging from 400 to 4000 cm^−1^, with a precision of ±0.05. As the self-assembly time increased, the $\kappa_{\lambda }$ increased rapidly in the wavenumber range of 400–1200 cm^−1^ while increased slowly in the wavenumber range of 1600–4000 cm^−1^ with small change ranges of 0.3–0.55. It should be noted that TE#2 and TE#3 presented nearly comparable values in the wavenumber range between 2000 and 2800 cm^−1^. This indicates a maximum index for $\kappa_{\lambda }$ at these wavenumbers. Therefore, it is an effective strategy to increase the optical $\kappa_{\lambda }$ of the TiO_2_-coated electrospun fibers through controllable self-assembly time.

The change in $n_{\lambda }$ seems insignificant at wavenumbers of 800–4000 cm^−1^. For instance, there was only a 0.1 increase in $n_{\lambda }$ at 1200 cm^−1^ as the self-assembly cycles increased from zero to six. However, a remarkable difference in $n_{\lambda }$ could be seen at the wavenumber of 400 cm^−1^. The $n_{\lambda }$ value at 400 cm^−1^ increased from 2.30 for PE to 2.50, 2.75, and 2.85 for TE#1, TE#2, and TE#3, correspondingly. Therefore, at some wavenumbers, the optical $n_{\lambda }$ of TiO_2_-coated electrospun fibers can be effectively increased through controllable self-assembly time.

Concerning a coupling optical effect of absorption and scattering of fibrous materials, the extinction factor ($Q_{\lambda }$) of the TiO_2_-coated electrospun fibers can be calculated as follows

(8) $$Q_{\lambda }=\frac{\pi d_{f}In\left(\tau_{\lambda }\right)}{4L_{m}\upsilon_{f}}$$

Table 2 gives the $Q_{\lambda }$ of PE and TE#1–TE#3 for wavenumbers ranging from 400 to 4000 cm^−1^, with a precision of ±0.005. It can be observed that at most wavenumbers, the $Q_{\lambda }$ increased with an increase in self-assembly cycles. This indicates an enhancement in the overall capacity of optical extinction after the TiO_2_ assembly process. However, at some wavenumbers, there was a maximum in $Q_{\lambda }$ for TE#2 obtained from four self-assembly cycles. For instance, the $Q_{\lambda }$ at 1200 cm^−1^ increased from 0.665 for PE to 0.850 for TE#2, while the corresponding value of TE#3 was 0.820. This was due to a similar decrease of refractive index, as the refractive index decreased from 1.70 of TE#2 to 1.65 of TE#3 at 1200 cm^−1^ (Table 1). Therefore, after controllable self-assembly, the optical $Q_{\lambda }$ of TiO_2_-coated electrospun PVDF fibers was effectively increased.

### 3.3. Controllable Self-Assembly for Increasing Optical Extinction Capacity of TiO_2_-Coated Electrospun Fibers

With the aim to further predict surface engineering influence by considering the volume and fiber diameter of fibrous membranes concerning infinite cylindrical fibers with an axis vertical to the radiation heat flux, the spectral extinction coefficient ($\beta_{\lambda }$, cm^−1^) can be calculated by

(9) $$\beta_{\lambda }=4Q_{\lambda }\cdot v_{f}/\pi d_{f}$$

**Figure 8:**
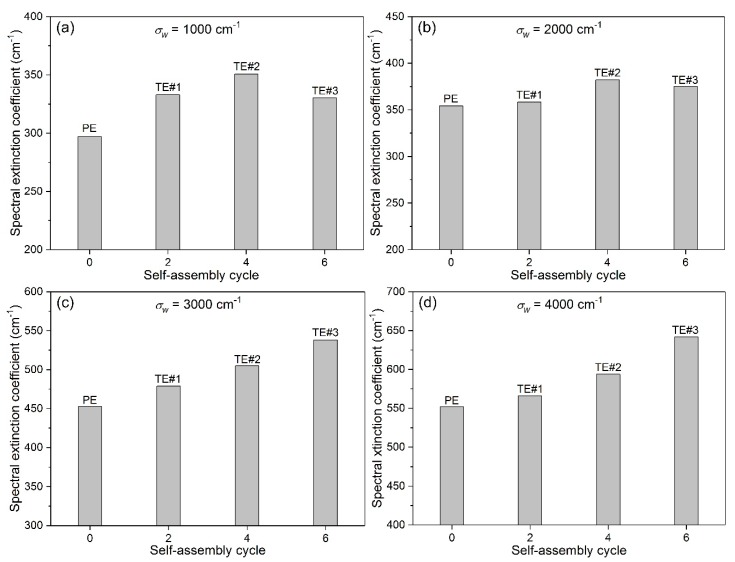
$\beta_{\lambda }$ for various self-assembly cycles wavenumbers of (**a**) 1000 cm^−1^; (**b**) 2000 cm^−1^; (**c**) 3000 cm^−1^, and (**d**) 4000 cm^−1^.

Figure 8a–d show the $\beta_{\lambda }$ of PE and TE#1–TE#3 at wavenumbers of 1000, 2000, 3000, 4000 cm^−1^, respectively. From Figure 8a, it can be observed that with an increase in the self-assembly time, the $\beta_{\lambda }$ increased first, then kept stable with a maximum. A similar existence of the maximum $\beta_{\lambda }$ with an increase in the self-assembly time could also be seen in Figure 8b. It is possible that with an increase in the self-assembly cycles, the porosity of the TiO_2_ nanolayer decreases, and thereby decreases the scattering of small particles, resulting in the decline of the overall optical extinction capacity. Furthermore, there was a negative correlation between the $\beta_{\lambda }$ value and the fiber diameter based on Equation (9). Obviously, TE#3 had a higher average fiber diameter than TE#2. 

The $\beta_{\lambda }$ values in Figure 8c,d obviously increased with an increase in the self-assembly time at wavenumbers of 3000 and 4000 cm^−1^. Therefore, it is an effective strategy to increase the optical $\beta_{\lambda }$ of TiO_2_-coated electrospun fibers through controllable self-assembly time. Moreover, the $\beta_{\lambda }$ increased with an increase in the wavenumber. For instance, as the wavenumber increased from 1000 to 4000 cm^−1^, the $\beta_{\lambda }$ value of TE#3 increased from 330 to 640 cm^−1^, which can be explained by the refractive index at high wavenumbers being presented as much greater than that at low wavenumbers, as shown in Table 1.

Unlike Figure 8c,d, it was found that the $\beta_{\lambda }$ values in Figure 8a,b first increased, then decreased with an increase in the self-assembly time. It is possible that as the self-assembly time increased from four to six, the porosity of the TiO_2_ layer is decreased, which thereby decreased the overall scattering capacity. Another reason may lie in the negative correlation between the $\beta_{\lambda }$ value and the fiber diameter based on Equation (9). Obviously, TE#3 had an average fiber diameter that was greater than TE#2.

It should be noted that the $\beta_{\lambda }$ values at wavenumbers of 1000 and 2000 cm^−1^ had different trend from those at 3000 and 4000 cm^−1^; the former showed a maximum at TE#2 while the latter increased from TE#2 to TE#3. It is likely that the lower wavenumber presents a greater influence on the increase of $\beta_{\lambda }$. This is consistent with the transmittances in Figure 7a, where TE#2 showed even smaller transmittances than TE#2 at lower wavenumbers such as 1000 and 2000 cm^−1^.

For the purpose of quantitatively evaluating the influence of the self-assembly time on the $\beta_{\lambda }$ of the TiO_2_-coated electrospun fibers, an increment percentage of the spectral extinction coefficient ($P_{\beta ,\lambda }$) was introduced and calculated by

(10) $$P_{\beta ,\lambda }=\frac{\beta_{\lambda }\left(n_{L}\right)-\beta_{\lambda }\left(0\right)}{\beta_{\lambda }\left(0\right)}\times 100\%$$

**Figure 9:**
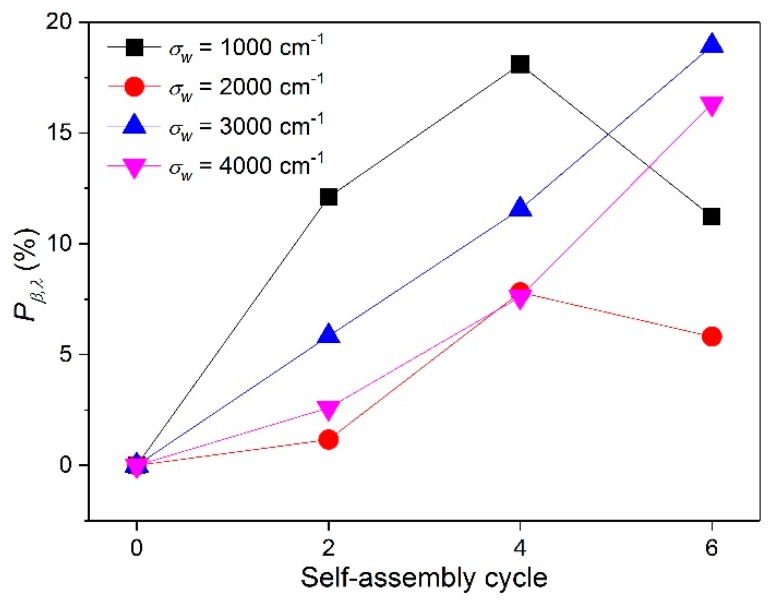
Increment percentage of spectral extinction coefficient at different wavenumbers.

Figure 9 shows the $P_{\beta ,\lambda }$ for various self-assembly cycles at wavenumbers of 1000, 2000, 3000, and 4000 cm^−1^, respectively. It was observed that $P_{\beta ,\lambda }$ increased at most wavenumbers with an increase in the self-assembly time. For instance, these percentages at wavenumbers of 1000, 2000, 3000, and 4000 cm^−1^ were 18.1%, 7.8%, 11.6%, and 7.6% for TE#2, which were much greater than the 12.1%, 1.2%, 5.8%, and 2.6% for TE#1, respectively. Therefore, the $\beta_{\lambda }$ could be effectively enhanced by increasing the self-assembly time.

Considering the spectral extinction coefficient for a wavenumber range of 400–4000 cm^−1^, an optical parameter that indicates the overall optical enhancement should be introduced such as the Rosseland extinction coefficient ($\beta_{T}$). The $\beta_{T}$ represents a recombination of the spectral extinction coefficient by coupling the variations of the wavelength and surrounding temperature, which can be calculated by

(11) $$\beta_{T}={\left(\overset{\infty }{\underset{0}{\int }}\frac{1}{\beta_{\lambda }}\frac{\partial E_{b\lambda }}{\partial E_{b}}d\lambda \right)}^{-1}$$

where $E_{b}$ represents the emissive power of black body, and $E_{b\lambda }$ represents the spectral emissive power of black body.

**Figure 10:**
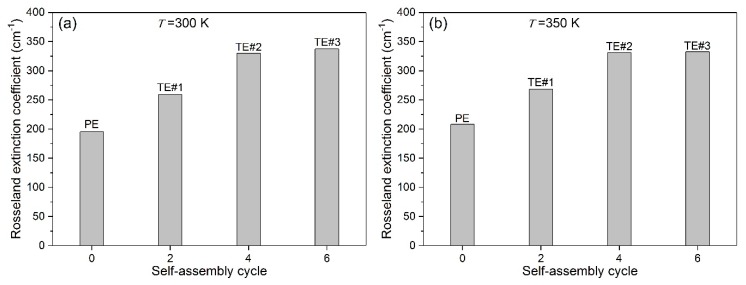

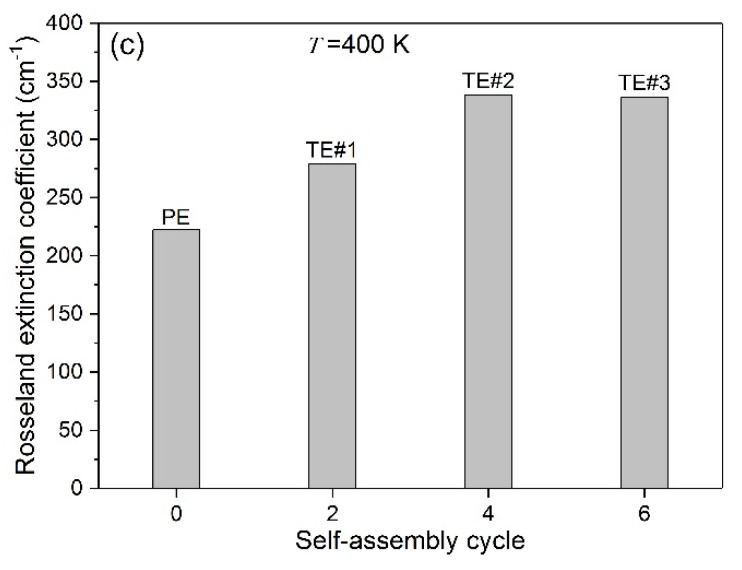
Rosseland mean extinction coefficient for various self-assembly cycles at (**a**) 300 K; (**b**) 350 K, and (**c**) 400 K.

Figure 10a–c show the predicted results of $\beta_{T}$ for PE and TE#1–TE#3 at temperatures of 300, 350 and 400 K, respectively. It can be observed from Figure 10a that at 300 K, the $\beta_{T}$ increased rapidly from 195 to 330 cm^−1^ as the self-assembly time increased from zero to six. In comparison, the $\beta_{T}$ increased from 205 to 335 cm^−1^ at 350 K, and from 220 to 340 cm^−1^ at 400 K. It is worth noting that the $\beta_{T}$ result was quite different from the spectral extinction coefficient, which may appear to decrease as the self-assembly time increased from four to six at wavenumbers of 1000 and 2000 cm^−1^ because the $\beta_{T}$ represents an overall result by integrating all the wavenumbers of 400–4000 cm^−1^.

It should be noted that the $\beta_{T}$ increased slightly or even approximately equal for TE#4 and TE#6 at different temperature. This indicates that a maximum enhancement of $\beta_{T}$ could be obtained with an increase in the self-assembly time. Therefore, controllable self-assembly could be sufficient for the increase in the optical extinction capacity of TiO_2_-coated electrospun fibers.

With the aim to further evaluate the influence of the self-assembly time on the $\beta_{T}$ of TiO_2_-coated electrospun fibers, an increment percentage of the Rosseland extinction coefficient ($P_{\beta ,T}$) was introduced and calculated by

(12) $$P_{\beta ,T}=\frac{\beta_{T}\left(n_{L}\right)-\beta_{T}\left(0\right)}{\beta_{T}\left(0\right)}\times 100\%$$

**Figure 11:**
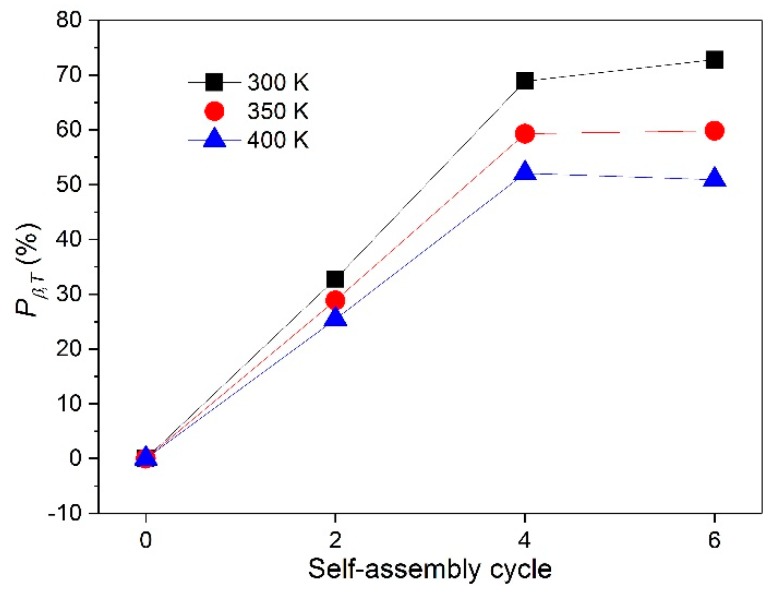
Increment percentage of Rosseland extinction coefficient.

Figure 11 shows the $P_{\beta ,T}$ for various self-assembly cycles at temperatures of 300, 350, and 400 K. It can be observed that the $P_{\beta ,\lambda }$ increased rapidly as the self-assembly time increased from zero to four. For instance, these percentages at 300, 350, and 400 K were 69%, 59%, and 52%, respectively, for TE#2, while the corresponding values for TE#1 were only 33%, 29%, and 25%, respectively.

$P_{\beta ,\lambda }$ increased slightly or even kept stable when the self-assembly time was greater than two. For instance, as the self-assembly cycles increased from four to six, the $P_{\beta ,\lambda }$ varied from 69%, 59%, and 52% to 72%, 60%, and 51% at 300, 350, and 400 K, respectively. This indicates that a further assembly time from four to six may exert an insignificant influence on the optical property. Overall, through assembling TiO_2_ nanoparticles, the increment percentage of $\beta_{T}$ could reach as high as approximately 50–70%.

In order to further compare the influence surface morphologies on the infrared extinction of cylindrical fibers, five surface morphologies for increases in the infrared extinction were summarized in Table 3 and shows that a dense and smooth distribution of TiO_2_ presented an increase in the infrared extinction capacity with 5000 cm^−1^ [24], which was greater than that of the random distribution of TiO_2_ with 17 m^2^·kg^−1^ (~17 cm^−1^) by direct doping [1,39]. This is reasonable because a coating thickness of ~300 nm is a much larger amount than a content 20 wt. %. A TiO_2_ nanorod is a special doping mode on the fiber surface, however, it only presented an increase of 7.3–10 m^2^·kg^−1^ in infrared extinction [26], which was smaller than that of the TiO_2_ random distribution. It is possible that the TiO_2_ nanorod was ~200 nm in diameter and 1.5 μm in length, which is inferior to a diameter of 3.5 μm in terms of infrared extinction [22].

It should be noted that the TiO_2_ rice-like distribution was reported to be a highly efficient coating mode because it highly increased the specific surface area of the coating layer with a TiO_2_ size of ~30–50 nm [27]. The increase in the infrared extinction could be as high as 195 cm^−1^. However, this method consumed a large amount of nano TiO_2_ with a thickness of ~100–200 nm. In comparison, the self-assembly mode consumed a small amount of nano TiO_2_ to achieve a considerable increase in the infrared extinction capacity of fibers because the coating layer composed of TiO_2_ with a nano size of ~7–10 nm could be highly porous with a significantly increased specific surface area by self-assembly. For instance, the increase of infrared extinction presented as high as ~120–130 cm^−1^ by incorporating only 5.7 wt. % of assembled TiO_2_.

There was a coupling mechanism between coating content, TiO_2_ size, and TiO_2_ particle content, which could determine the increase of infrared extinction of the TiO_2_ surface coating. A greater coating content could be conducive to a greater increase in the infrared extinction. The optimal size of TiO_2_ particles for maximum infrared extinction is 3.5 μm, and the extinction decreases away from the optimal value [40]. However, the particle content of the porous TiO_2_ layer seems more likely be neglected according to previous studies [41], which is likely to be due to a lack of a measure to obtain the uncountable TiO_2_ particles. In fact, the particle number can be reflected by the porosity and specific surface area, and normally a larger particle number contributes to a greater infrared extinction.

Therefore, a high optical extinction of electrospun fiber could be expected through the controllable self-assembly of nano TiO_2_ with an extremely high porosity and specific surface area. The better optical properties of the TiO_2_-coated electrospun PVDF fiber obtained from controllable self-assembly in this work can be attributed to a higher increase in the light-scattering and reflecting ability than the other four surface morphologies as above-mentioned.

## 4. Conclusions

This paper presented the engineering surface and optical properties of TiO_2_-coated electrospun PVDF fibers through controllable self-assembly. By controlling self-assembly time, four samples, i.e., PE and TE#1–TE#3 with different self-assembly cycles, were experimentally obtained via electrospinning and LBL self-assembly techniques. Based on measurements and characterizations, the surface roughness and the average diameter of TiO_2_-coated electrospun PVDF fibers increased with an increase in the self-assembly time. Concerning the TiO_2_ coating layer, with an increase in the self-assembly time, the thickness and the assembled content increased while the porosity decreased. By controlling the self-assembly time, the optical scattering and absorption properties of TiO_2_-coated PVDF electrospun fibers were effectively increased. Through controllable self-assembly, the infrared extinction could be increased from 205–220 cm^−1^ for PE to 335–340 cm^−1^ for TE#3, which accounts for an enhancement of almost 50–75%. We believe that the controllable self-assembly has the potential to be one of the most efficient coating modes to increase the infrared extinction given its achievement of a highly porous coating structure.

## Figures and Tables

**Table 1:** $\kappa_{\lambda }$ and $n_{\lambda }$ of PE and TE#1–TE#3 at 400–4000 cm^−1^.

Wavenumber (cm^−1^)	Absorption Index ($\kappa_{\lambda }$)				Refractive Index ($n_{\lambda }$)			
	PE	TE#1	TE#2	TE#3	PE	TE#1	TE#2	TE#3
400	0.25	0.30	0.45	0.50	2.30	2.50	2.75	2.85
800	0.50	0.65	0.75	0.85	2.15	2.25	2.25	2.20
1200	1.15	1.50	1.55	1.60	1.60	1.60	1.70	1.65
1600	0.30	0.40	0.40	0.40	1.60	1.60	1.60	1.60
2000	0.35	0.40	0.45	0.40	1.60	1.55	1.60	1.60
2400	0.35	0.35	0.40	0.40	1.60	1.50	1.55	1.60
2800	0.35	0.35	0.40	0.35	1.55	1.50	1.55	1.60
3200	0.35	0.35	0.40	0.40	1.50	1.50	1.50	1.55
3600	0.35	0.35	0.40	0.45	1.45	1.45	1.45	1.50
4000	0.40	0.40	0.50	0.55	1.40	1.40	1.40	1.40

**Table 2:** $Q_{\lambda }$ of PE and TE#1–TE#3 at 400–4000 cm^−1^.

Wavelength (cm^−1^)	Extinction Factor ($Q_{\lambda }$)			
	PE	TE#1	TE#2	TE#3
400	0.065	0.105	0.165	0.195
800	0.290	0.405	0.505	0.515
1200	0.665	0.770	0.850	0.820
1600	0.310	0.400	0.470	0.455
2000	0.455	0.520	0.600	0.580
2400	0.545	0.585	0.680	0.685
2800	0.640	0.640	0.760	0.785
3200	0.700	0.725	0.865	0.930
3600	0.765	0.790	0.925	1.000
4000	0.835	0.875	1.015	1.150

**Table 3:** Different surface morphologies for increases in infrared extinction.

Surface Morphology (TiO_2_)	Structure Schematic	Coating Technique	Size of Coating Particles	Coating Content (or Thickness)	Increases in Infrared Extinction
[39] Random. Reproduced with permission from [1] Elsevier, 2008.	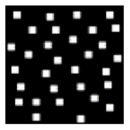	Direct doping	~3.5 μm	Content 20 wt. %	By 17 m^2^·kg^−1^
Dense and low roughness. Reproduced with permission from [24]. Elsevier, 2004.	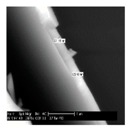	Soaking and dipping	None	Thickness ~290–320 nm	By 5000 cm^−1^ at axis direction
Nanorods. Reproduced with permission from [26]. Elsevier, 2012.	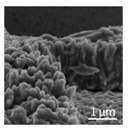	Seed-hydrothermal	~200 nm in diameter, 1.5 μm in length	Content ~8.4–21.6 wt. %	By ~7.3–10 m^2^·kg^−1^
Rice-like. Reproduced with permission from [27]. Elsevier, 2010.	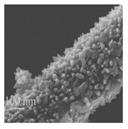	Ultrasonic-assistance	~30–50 nm	Thickness ~100–200 nm, large content	By ~46–195 cm^−1^
Rough and continuous	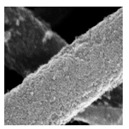	Self-assembly	~7–10 nm	Thickness 31 nm, content 5.7 wt. %	By ~120–130 cm^−1^
